# Metaproteomics as a Complementary Approach to Gut Microbiota in Health and Disease

**DOI:** 10.3389/fchem.2017.00004

**Published:** 2017-01-26

**Authors:** Bernardo A. Petriz, Octávio L. Franco

**Affiliations:** ^1^Department of Health, Molecular and Physiologic Adaptations to Exercise, Centro Universitário do Distrito FederalBrasília, Brazil; ^2^S-Inova Biotech, Universidade Católica Dom BoscoCampo Grande, Brazil

**Keywords:** mass spectrometry based proteomics, fecal metaproteome, gut microbiota, metabolomics, microbiota genome catalog, OMICS, LC-MS/MS

## Abstract

Classic studies on phylotype profiling are limited to the identification of microbial constituents, where information is lacking about the molecular interaction of these bacterial communities with the host genome and the possible outcomes in host biology. A range of OMICs approaches have provided great progress linking the microbiota to health and disease. However, the investigation of this context through proteomic mass spectrometry-based tools is still being improved. Therefore, metaproteomics or community proteogenomics has emerged as a complementary approach to metagenomic data, as a field in proteomics aiming to perform large-scale characterization of proteins from environmental microbiota, such as the human gut. The advances in molecular separation methods coupled with mass spectrometry (e.g., LC-MS/MS) and proteome bioinformatics have been fundamental in these novel large-scale metaproteomic studies, which have further been performed in a wide range of samples including soil, plant and human environments. Metaproteomic studies will make major progress if a comprehensive database covering the genes and expresses proteins from all gut microbial species is developed. To this end, we here present some of the main limitations of metaproteomic studies in complex microbiota environments, such as the gut, also addressing the up-to-date pipelines in sample preparation prior to fractionation/separation and mass spectrometry analysis. In addition, a novel approach to the limitations of metagenomic databases is also discussed. Finally, prospects are addressed regarding the application of metaproteomic analysis using a unified host-microbiome gene database and other meta-OMICs platforms.

## Introduction

The use of mass spectrometry in biomolecular identification is responsible for incalculable progress in the biomedical and health field. This progress is directly related to the significant technologic improvement in these detection tools concerning qualitative and quantitative aspects (Aebersold and Mann, 2003). In this context, proteome-wide scale analysis may be considered one of the main tools in disease biomarker identification (Zhang A. et al., 2016). This is clearly observed in several tissue targets (e.g., cardiac, skeletal muscle, adipose and hepatic tissue, as well as body fluids, blood, urine, and saliva) in health and disease-related fields, such as physical activity, cancer, cardiovascular and metabolic disturbance (Diamandis, 2004; Hu et al., 2005; Mira-Pascual et al., 2015; Petriz et al., 2015; Savas et al., 2016; Thomas et al., 2016).

In the past few years, metagenomic studies have linked the gut microbiota to the pathogenesis of obesity. In this context, several mechanisms were investigated, including enhanced energy extraction from complex polysaccharides during digestion, SCAFS production with direct effect on the energy-homeostasis center in the hypothalamus axis and the stimulation of pro-inflammatory signaling (Turnbaugh et al., 2006). Some of these obesogenic factors are thought to occur by the interaction of selective microbiota community with gut genes, influencing the host metabolism. Thus, gut transcriptome studies have brought some interesting insights to this aspect, also revealing novel candidates linking microbiota to obesity and cardiovascular disease (Ussher et al., 2013; Tang and Hazen, 2014; Gregory et al., 2015; Li et al., 2015; Aron-Wisnewsky and Clément, 2016).

Besides the increasing data linking obesity, type 2 diabetes (Cai et al., 2015; Forslund et al., 2015) and auto-immune disorders, such as diabetes type I (Burrows et al., 2015) to alterations within the gut microbiota, recent studies have also associated lower levels of certain bacterial families (e.g., Veillonellaceae) with increased blood pressure. This was observed in salt-sensitive and Dahl salt-resistant rat strains, pointing to some new insights in the interaction of host-gut microbiota and the regulation of blood pressure (Mell et al., 2015). In addition, the progression of cardiovascular risk factors, such as atherosclerosis has also been linked to gut microbiota (Howitt and Garrett, 2012; Ussher et al., 2013; Gregory et al., 2015). Thus, further conclusions concerning this aspect are still limited.

The various OMICs approaches have led to great progress linking the microbiota profile to health and disease. However, the investigation of this area through proteomic mass spectrometry-based tools is still being improved. As a result, there is a large gap in this field, where the identification of gene products from the gut's microbiota will lead to meaningful and complementary information about the relationship between host-microbiota and the outcomes of this close interaction in health and in the pathogenesis of complex diseases, such as obesity and cardiovascular disorders.

Taking this into consideration, in this review, we present the application and the main limitations of metaproteomic studies in complex microbiota environments, such as the gut, also addressing the up-to-date pipelines in sample preparation prior to fractionation/separation and mass spectrometry analysis. In addition, a novel approach to the limitations of metagenomic databases is also discussed. Finally, the prospects for the application of metaproteomic analysis using a unified host-microbiome gene database are addressed.

## From metagenome to metaproteome analysis of gut microbiota

Considering the gastrointestinal tract as one of the most complex biological ecosystems ever studied, high-throughput metagenomic studies (e.g., 16S rRNA profiling) have done a good job in delivering some interesting insights on the compositions of the host microbial communities, and their effect on host health and disease status (Round and Mazmanian, 2009). Due to these studies, intestinal microbes are linked to key roles in host defense, including their action against pathogen invasion, innate immune regulation and inflammatory responses, nutrient processing, and energy balancing through metabolic process. Also, an unbalanced microbiota profile may be related to metabolic disorders, such as obesity and diabetes (Ley et al., 2006; Ridaura et al., 2013), and even cardiovascular disease, such as atherosclerosis (Gregory et al., 2015; Li et al., 2015).

However, these classic phylotype profiling studies are limited to the identification of microbial constituents, where poor information is known about the molecular interaction of these bacterial communities with the host genome and the possible outcomes in host biology. It is possible that this gap had influenced the development of complementary and more sensitive tools to better investigate the entire scene. The study of the environmental proteome seemed to fulfill this gap, also leading to significant clinical potential (Haange and Jehmlich, 2016). Compared to metagenomics and metatranscriptomics, the major positive aspect of metaproteomics relies on “function” information. Identification of proteins and their assignment to specific taxa and how they interact within the host are key elements in better understanding the host physiology under physiologic and pathologic conditions. Thus, after a decade, the technical aspects of this analysis are still a major challenge to the field (Wilmes et al., 2015).

Considering the host gut-proteome as a fundamental element for maintaining the mutualistic relationship, also reporting on the status of the interaction of host-to-microbiota, it is very important to identify and address the function of as many proteins encoded by the gut microbes as possible. This is a key factor to enhance our understanding of the host-microbe interactions in the gut ecosystem. Also, from this perspective, novel complementary treatment for metabolic disorders and even inflammatory bowel disease may be formulated. Therefore, metaproteomics or community proteogenomics has emerged as a complementary approach to metagenomic data, as a field in proteomics aiming to perform large-scale characterization of proteins from environmental microbiota, such as the human gut (Wilmes and Bond, 2004).

In the past decade, this approach evolved from few proteins uncovered by classic 2-DE and *de novo* sequencing to thousands of proteins resolved by shotgun LC-MS/MS permitting simultaneous protein separation, quantification and identification (Wilmes et al., 2015). The advances in molecular separation methods coupled with mass spectrometry (e.g., LC-MS/MS) and proteome bioinformatics were fundamental to these novel large-scale metaproteomic studies, which have further been performed in a wide range of samples from soil, to plant and human environments (Püttker et al., 2015; Xiong et al., 2015a; Zampieri et al., 2016). However, it must be considered that the complexity of environmental samples is higher than the classic proteomics studies, owing to the lack of a well-known genome database.

Despite the great advances in this field in the past decade, the characterization of the microbiome proteome presents a high level of complexity, especially when considering the gastrointestinal environment. It is estimated that in fecal samples with approximately >21,000 taxa, there are more than 63,000,000 unique proteins (Wilmes et al., 2015), of which 2900 were identified as the host-microbiota signature of Crohn's disease (Erickson et al., 2012). Besides the high complexity of fecal and gut microbial content, severe proteolytic events in the gastrointestinal tract may also affect its analysis (Lichtman et al., 2013). Thus, recently, major progress has been made to overcome these methodological limitations.

## Metaproteomics in gut microbiota

In the gastrointestinal environment, metaproteomic studies were initially applied to investigate the microbial mucosa-lumen interface in several intestinal *loci* (Li et al., 2011), as done in the comparison of healthy and inflamed mucosa in an inflammatory bowel disease study (Presley et al., 2012). Others have found significant variation in intestinal epithelial barrier proteins between Crohn's disease patients and healthy individuals (Erickson et al., 2012). Thus, the analysis of gut metaproteome and metagenome are usually conducted with fecal samples, not only because they are easily accessible matrices that permit collections in several temporal points, but also because they present a great amount of biomass. Moreover, the analysis of fecal samples collected at different time-points may reflect the intestinal conditions under healthy and pathologic conditions, which has been extensively analyzed in metagenomic studies (Serino et al., 2012; Petriz et al., 2014).

The first large-scale metaproteomic study of the human fecal sample identified up to 1340 non-redundant proteins (Verberkmoes et al., 2009). It was observed that 30% of the measured spectra were matched to the human protein database, indicating that the host proteome plays a significant role in fecal metaproteomics. The study of the fecal metaproteome is also being conducted to understand the process of gut colonization better, since metaproteomic data may provide more insights about the characterization of metabolic activity and host-microbe interaction, being a complement to infant metagenomic data (Morowitz et al., 2011; Sharon et al., 2013). In this sense, it must be considered that the presence of host-cells protein's and endogenous factors are some of the many challenging events in the analysis of the gut metaproteome (Xiong et al., 2015a). Furthermore, the preparation and optimization of the fecal sample prior to mass spectrometry (MS) is possibly essential for efficient metaproteomic analysis.

**Figure 1:**
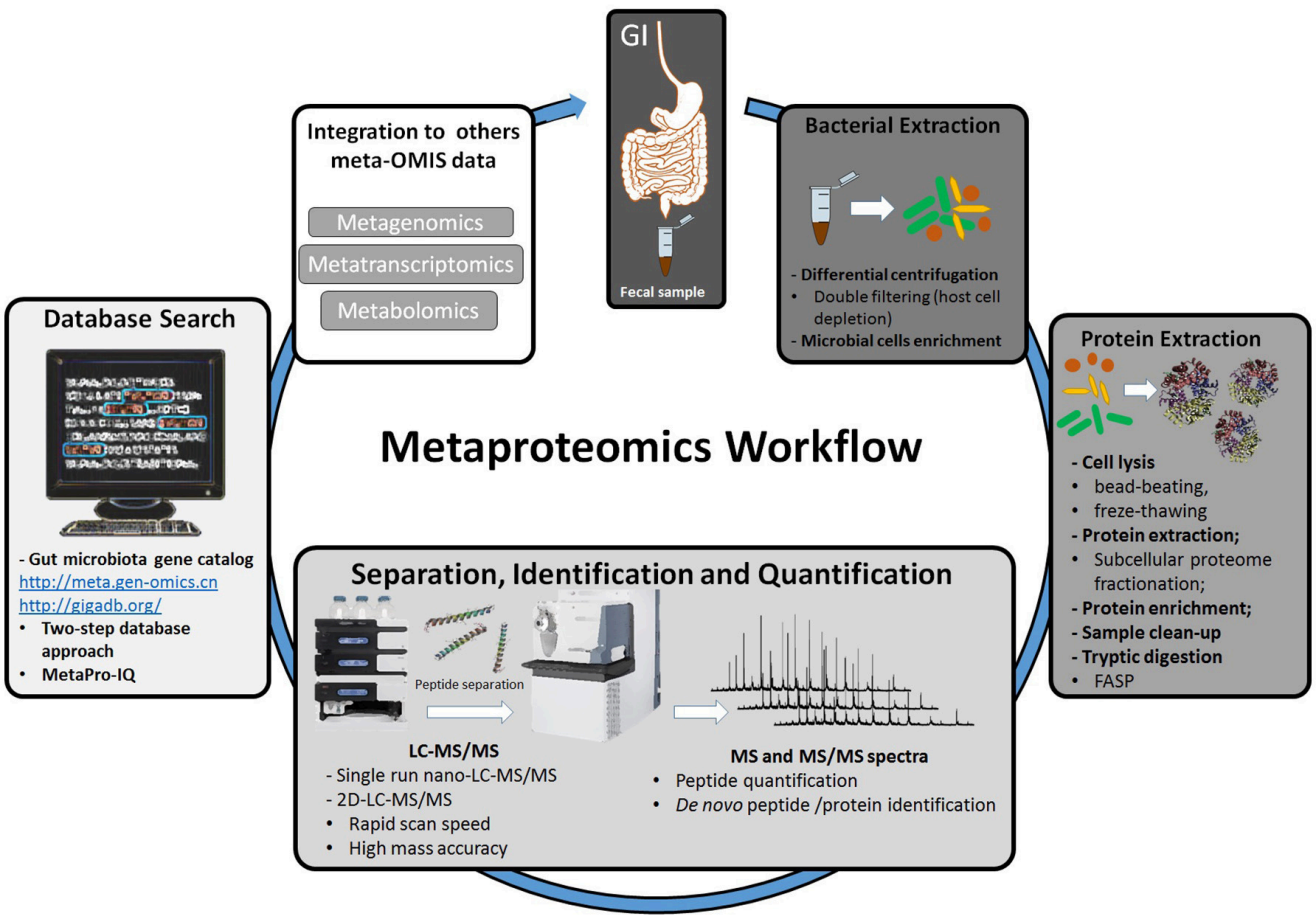
**Representation of a workflow in a metaproteomic analysis of fecal sample extracted from the gut, starting from the bacterial extraction process, followed by microbial protein extraction and enrichment, protein and peptide separation, identification, and quantification and MS/MS search against genome databases**. FASP; filtered-aided sample preparation.

Considering the complexity of metaproteomic studies applied to a wide range of microbial consortia, experts in the field have suggested a series of considerations for this field (for a full review see Wilmes et al., 2015; Smirnov et al., 2016). In a general way, a metaproteomic study performed by shotgun is conducted by considering three to four main steps, which include: (1) efficient extraction of proteins from the entire microbial community; (2) sample clean-up from chemicals used in the extraction process, usually detergents and other compounds that interfere with the enzymatic digestion and MS analysis; (3) depletion of host cells and the enrichment of microbial cells to avoid host-cell contamination (this step is usually used in complex samples, such as gastrointestinal content); (4) reduction of sample complexity through the pre-fractionation of proteins and peptides before MS analysis. Considerable attention must be paid to the extraction process, since the microbiota is composed of a wide range of microorganisms (e.g., Gram-negative and Gram-positive bacteria, fungi), and it is suggested that the depletion of species with higher resistance to lysis methods should be avoided. For example, it has been shown that the combination of bead-beating with freeze-thawing enhances protein extraction from yeast and Gram-positive bacteria (Tanca et al., 2014). A general metaproteomic analysis workflow is presented in Figure 1.

## Mass spectrometry analysis in fecal metaproteomics

Considering metaproteomics as a relatively novel analytical procedure, more effort is required to enhance and improve the process of sample preparation in the analysis of gut metaproteome. As mentioned in the previous section, in addition to the challenging aspects of fecal samples (e.g., the sample's large diversity in gut microbial composition, wide dynamic range of protein abundance and poor or insufficient genome information), fecal metaproteomic analysis is commonly impaired by the high number of host-cells and proteins, which overshadow the microbial proteome.

It was recently observed that microbial peptides that are co-eluted with dominant human peptides are ion suppressed, limiting their identification upon MS/MS analysis (Xiong et al., 2015b). To overcome part of these limitations, differential centrifugation is commonly used in fecal samples in order to enrich microbial cell content (Verberkmoes et al., 2009; Erickson et al., 2012). However, the amount of fecal sample is still a limiting issue. For this reason, a double filtering (DF) separation step was proposed by Xiong et al. (2015b) as an alternative to enrich microbial biomass, while depleting human cells and proteins prior to the separation and identification by 2D LC-MS/MS. Authors reported that this strategy successfully enriched low-abundant microbial proteins, also preserving the relative distributions of protein abundance in each analyzed sample.

In shotgun metaproteomics, the absence of available database for peptide spectra matching (PSM) is one the main limitations in the analysis of the gut metaproteome, also limiting cross-study comparisons. To date, customized databases composed of a series of unknown gut microbial genome are often used (Erickson et al., 2012; Kolmeder et al., 2012) instead of high sequence coverage databases (e.g., NCBI-nr) because of their time-consuming process and less sensitive peptide identifications when using false discovery rate filtering (FDR) (Jagtap et al., 2013). However, it must be considered that these synthetic databases present some limitations, because a number of intestinal microbes are uncultivable with un-sequenced genomes. In the best scenario, functionally annotated metagenomes derived from whole genome sequencing from the same sample would enhance the protein identification in metaproteomic studies. This approach was used in the study of Kolmeder et al. (2012), where metagenome and single genome sequence data from thousands of mass spectra from each sample generated ~1000 peptides per sample, indicating also ~1000 core proteins per sample. Thus, when this is not an option, a matched metagenome database is suggested as an alternative to manual customized databases.

It has been shown that the sensitivity of peptide and protein identification was enhanced by a two-step approach, in which a target-only database was primarily used to generate a narrower database, followed by second target-decoy database research (Jagtap et al., 2013). Thus, major progress has been made with the combination of a matched metagenome database with the two-step approach, where 13,000 peptides, corresponding to 3000 proteins were identified from mice cecum samples (Tanca et al., 2014). Here an optimized pipeline, including sample extraction by bead-beating and freeze-thawing, sample cleanup and digestion by filtered-aided sample preparation (FASP) and a single run nanoLC-MS/MS was proposed as a straightforward method for metaproteome analysis (Tanca et al., 2014). It is suggested that single run nanoLC-MS/MS is a less laborious and time-consuming approach compared to two-dimensional LC-tandem mass spectrometry (MS/MS) (Schneider and Riedel, 2010). It has also been suggested that the approach for protein identification and analysis leads to significant impacts on metaproteomic data (Tanca et al., 2013). Thus, the pipeline proposed by Tanca et al. (2014) was tested on two samples of murine fecal microbiota with separate LC-MS/MS analysis being conducted for each fecal sample. The obtained MS spectra were searched against a matched metagenomic database with archived ORFs (open reading frames) from all experimental sequencing from the entire fecal metagenome, including mouse and soybean proteome and the sequences from fungal and archaeal. This proposed platform was shown to be more time-effective than 2D-LC-MS/MS, also presenting an in-depth characterization of gut microbiota by uncovering proteins from over 600 different microbial species and 250 functional protein families.

Metaproteomic studies will make major progress if a comprehensive database covering the genes and expresses proteins from all gut microbial species is developed. As an example, a recent study used the well-annotated human (9.9 Gut million genes) (Li et al., 2014) and mouse (2.6 million genes) (Xiao et al., 2015) gut microbial gene database (available at: http://gigadb.org/) to set an improved and universal approach for metaproteomic identification and quantification (MetaPro-IQ) (Zhang X. et al., 2016). MetaPro-IQ analysis led to the quantification of ~120,000 peptides, corresponding to 30,000 protein groups. A significant improvement in database limitations was also addressed in the recent study of Chatterjee et al. (2016) where the authors designed a scalable set of sequence databases. Thence, the authors developed a metagenomic analysis method (ComPIL) by integrating three protein sequencing databases (ProtDB, MassDB and SeqDB) with a proteomic search engine (Blazmass) for rapid matching of MS MudPIT spectra. Using this method, intracellular and secreted microbial proteins from five human stool samples in three technical replicates were identified on an average of more than 9000 protein loci per sample. Lastly, metaproteomic studies are mostly conducted in *in vivo* experiments. However, it must be considered that *ex vivo* gastrointestinal model systems (e.g., tissue cultures extracted from colon or rectum Grivel and Margolis, 2009) can also be used to validate and obtain more mechanistic information about the relationship and interaction between the host and its microbiota (Fritz et al., 2013). This was observed recently, when Tsilingiri et al. (2012) used this approach to demonstrate the effect of probiotics and postbiotics on the inflammatory properties of *Salmonella*.

## Prospects

In comparison to metagenomics and metatranscriptomics, the study of the metaproteome is still restricted and far from its full potential. Nevertheless, it is a consensus that metaproteomics delivers a great amount of valuable data for responding to diverse biologic questions concerning the host biology in health and disease. It is also notable that this approach still faces some technical challenges (e.g., sample preparation and analytical data acquisition and quantification). In order to reach its expected potential, these emerging technologies should be improved in order to reduce the wide dynamic ranges of different metaproteomics, also focusing on detecting methods for protein modifications and the integration of the meta-omics platforms for in-depth characterization of diverse microbial communities (Wilmes et al., 2015).

As occurred with metagenomics and metatranscriptomics, rapid technical advance is expected in the upcoming years to lower the cost of metaproteomics and routinely pair it up with the other meta-OMICs data in the study of gut microbiota, which also include the metabolomics approach (Smirnov et al., 2016). As an integrated approach to metaproteome, metabolomics has also delivered a comprehensive analysis of several biological sources (e.g., tissue, plasma, urine, faces), in order to track and monitor metabolites from the host and its microbes, and their co-metabolites (Palau-Rodriguez et al., 2015). However, in fecal samples, metabolomics is performed in other to track metabolites originating from the host, its microbes and food components, which may be an extremely useful tool for investigating metabolic disorders in the host and in relation to *in situ* alterations (Smirnov et al., 2016).

Still, as shown recently by Smirnov et al. (2016) an ISI Web of Science search indicated that the majority of publications concerning fecal metabolomics are attributed to disease-related issues, such as inflammatory bowel disease, cancer, infection, and obesity. In addition, this search indicated that when compared to 16S sequencing, metagenomics, and metabolomics, the field of metaproteomics is less explored, with fewer publications, indicating a vast field to be researched.

## Author contributions

All authors listed, have made substantial, direct and intellectual contribution to the work, and approved it for publication.

## Funding

Research funded by CNPq Brazil-PDJ Scholarship (152817/2016-6).

### Conflict of interest statement

The authors declare that the research was conducted in the absence of any commercial or financial relationships that could be construed as a potential conflict of interest.
